# Shifting mirrors: adaptive changes in retinal reflections to winter darkness in Arctic reindeer

**DOI:** 10.1098/rspb.2013.2451

**Published:** 2013-12-22

**Authors:** Karl-Arne Stokkan, Lars Folkow, Juliet Dukes, Magella Neveu, Chris Hogg, Sandra Siefken, Steven C. Dakin, Glen Jeffery

**Affiliations:** 1 Department of Arctic and Marine Biology, University of Tromsø, Tromsø, Norway; 2 Institute of Ophthalmology, University College London, 11-43 Bath Street, London EC1V 9EL, UK; 3 Moorfields Eye Hospital, London, UK

**Keywords:** seasonal adaptation, retina, scotopic vision

## Abstract

Arctic reindeer experience extreme changes in environmental light from continuous summer daylight to continuous winter darkness. Here, we show that they may have a unique mechanism to cope with winter darkness by changing the wavelength reflection from their tapetum lucidum (TL). In summer, it is golden with most light reflected back directly through the retina, whereas in winter it is deep blue with less light reflected out of the eye. The blue reflection in winter is associated with significantly increased retinal sensitivity compared with summer animals. The wavelength of reflection depends on TL collagen spacing, with reduced spacing resulting in shorter wavelengths, which we confirmed in summer and winter animals. Winter animals have significantly increased intra-ocular pressure, probably produced by permanent pupil dilation blocking ocular drainage. This may explain the collagen compression. The resulting shift to a blue reflection may scatter light through photoreceptors rather than directly reflecting it, resulting in elevated retinal sensitivity via increased photon capture. This is, to our knowledge, the first description of a retinal structural adaptation to seasonal changes in environmental light. Increased sensitivity occurs at the cost of reduced acuity, but may be an important adaptation in reindeer to detect moving predators in the dark Arctic winter.

## 1. Introduction

Animals living above the Arctic Circle experience extensive annual periods of permanent light and near-complete darkness in the summer and the winter sky, respectively. Winter, when the sun is below the horizon, has a deep blue hue owing to extensive Rayleigh scatter. This extreme photic environment poses unique challenges and one adaptive response in Arctic reindeer (*Rangifer tarandus*) has been the extension of their visual range into the near ultraviolet (UV) allowing greater use of winter light that is relatively UV rich [1]. This increases photon capture and matches sensitivity to the limited environmental light. In other respects, the reindeer retina appears to be largely similar to that found in other large ungulates [2].

An adaptive mechanism to dim illumination found in some mammals is the presence of a reflective surface behind the central retina, the tapetum lucidum (TL, commonly known as ‘cat's eye’) that resides under an unpigmented region of the retinal pigmented epithelium (RPE). This functions as a mirror providing the second opportunity for photon capture by reflecting light back through the retina, enhancing visual sensitivity. In mammals, it is predominately golden and is thought to represent an element of nocturnal evolutionary history. It had not been thought that this structure showed any adaptation to changes in environmental light [3,4].

Arctic reindeer possess a typical ungulate TL with a reflective surface composed of spaced collagen fibres (*tapetum fibrosum*) [3,4]. Given the extreme visual environment experienced by these animals, we have investigated whether this structure differs between the summer and winter solstices in the reindeer, and if so how this impacts on their retinal sensitivity.

## 2. Material and methods

### (a) Animals

Eurasian mountain reindeer (*Rangifer tarandus tarandus*) were used, originating from semidomesticated herds maintained by Sami pastoralists. Experiments were undertaken at the University of Tromsø, Norway (70° N). The sun is permanently above the horizon here from mid-May to the end of July and remains below it from late November to late January. Animals were of both sexes. They were 1.5–3 years old and maintained in large outdoor pens or bought directly from Sami herders in Troms and Finnmark Counties. All were maintained in the same way without seasonal dietary changes. They were killed with a head bolt gun and bled out. Eye collection and *in vivo* experimentation were undertaken during two weeks on either side of the summer and winter solstices. Analysis included measurements of TL total and spectral reflections, electron microscopic (EM) measurements of TL collagen spacing, electroretinogram (ERG) recordings, intra-ocular pressure measurements and measurements of pupil size. Ethical approval for all *in vivo* experiments was obtained from The Norwegian Animal Research Authority. In winter, a large proportion of the eyes were collected during routine slaughtering of reindeer.

### (b) *In vitro* measurements of reflections

#### (i) Measurements of total reflectance

Single eyes were collected from five winter and five summer unrelated animals and fixed with 10% formalin following enucleation at death. The cornea, lens and retina were removed revealing the TL. Reflectance measurements were undertaken 3 mm above the TL surface over an illuminated circular area approximately 2 mm in diameter. Measurements were made using an Ocean Optics USB2000 miniature fibre-optic spectrometer. This uses a grating to diffract the incoming light into a spectrum sensed by a 2048-element linear charge-coupled device-array (Ocean Optics, Dunedin, FL, USA). The absolute spectral response was calibrated using a calibrated tungsten halide lamp (Ocean Optics LS-1-CAL) and its spectral wavelength was calibrated using a Mercury–Argon source (Ocean Optics HG-1). An Ocean Optics fibre-optic reflection probe made up of seven optical fibres was used to make measurements. Six illuminating fibres hexagonally surrounded a single, central sensing fibre. The fibres were illuminated by the LS-1-CAL lamp. The sensing fibre was connected to the USB2000 spectrometer. Measurements were made relative to the reflectance produced by a flat white standard (Tintometer Ltd, Salisbury, UK) used to normalize all reflectance data as a percentage of the total. This was used before and after recording from each eye cup and each was recorded in triplicate. Measurements were focused on the central TL. To estimate the degree of scatter from the TL in summer and winter eyes, whole-mounted TL preparations were placed flat on a microscope slide and a micromanipulator used to hold the probe vertically less than 1 mm above the specimen. The specimen was dimly illuminated with 1.76 × 10^−3^ W m^−2^ at 510 nm which is similar to the max sensitivity of rhodopsin. Increased relative scatter should result in more light being detected in winter compared with that in summer in the sensing element of the probe. These values were selected to reflect the biological situation in winter.

#### (ii) Measurements of spectral reflection

Fourteen winter eyes and six summer eyes were collected and fixed as above, and the TL was cut free from peripheral tissues and maintained moist and photographed in raw format using a mounted Canon EOS digital camera with a 60 mm macro lens, such that the camera performed minimal data processing on the image. Illumination and distance were constant. There was no difference in TL area between different animals/seasons and images taken covered the full TL area. The default ungulate TL reflection is golden [3,4]. To confirm that any changes away from this were not owing to fixation, 10 eyes were examined by visual inspection fresh within 5 min of slaughter and during subsequent fixation. No change was seen.

Conversion from red, green, blue (RGB) images to hue, saturation and value (HSV) was done using the rgb2hsv command in the Matlab programming environment. Specifically, *R*, *G* and *B* values are scaled to be in the range 0–1.0 and the maximum and minimum values (*C*_max_ = max(*R*,*G*,*B*) and (*C*_min_ = min(*R*,*G*,*B*), respectively, used to calculate the range, *Δ* = *C*_max_ − *C*_min_. From these values, one can define the hue as
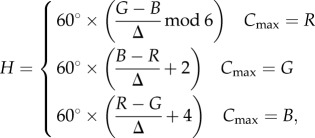
the saturation as
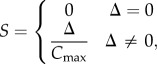
and the value as
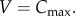


### (c) Electron microscopic measurements of collagen fibre spacing in the tapetum lucidum

Single eyes from four animals killed at the summer solstice and four animals killed at the winter solstice were enucleated and fixed as above for more than two weeks. Three of summer and three of winter eyes used here came from specimens used to examine total reflectance above. The cornea, lens and retina were removed. The central TL region just above the optic nerve head was dissected free in a block measuring approximately 5 × 5 mm containing the RPE through to the sclera. This was placed in 1% osmium tetroxide for 2 h and dehydrated through a graded series of alcohols before being embedded in araldite. Ultrathin sections were cut and examined under a transmission electron microscope. Photographs were taken of collagen fibres cut orthogonally at a magnification of ×20 000. The negatives from these were then projected onto a surface, increasing the magnification by a further ×400. The distance between 100 collagen fibres were measured on the projections from each eye.

### (d) Electroretinogram recordings

Animals were anaesthetized and maintained, and experiments undertaken as previously reported where full details are given [1]. ERG were recorded from the left eye of each anaesthetized animal, using a gold foil corneal electrode as the active electrode, ground and reference electrodes were subdermal platinum iridium needle electrodes. Eye position was maintained using a scleral suture, and the cornea was irrigated with saline and artificial tears. Proxymetacaine topical anaesthetic was used in addition to the general anaesthetic.

The stimulus was presented in an 18 cm diameter Ganzfeld bowl positioned over the eye and was generated using a range of light emitting diodes (LEDs), which were driven using a combination of pulse width and current modulation. For the lower intensity ranges, neutral density filters were used to further reduce the intensity. The white LEDs used had a spectral distribution from 420 to 620 nm.

Animals were dark adapted for more than 30 min before electrode application. A scotopic ERG intensity series was recorded to white light from subthreshold to the maximal light intensity available (more than 100 cd s^−1^ m^−2^). This spanned 9.5 log units of intensity. Animals were then light adapted with a white background of 25 cd m^−2^ for 10 min, and photopic transient and 30 Hz flicker ERGs were recorded to white light.

### (e) Pressure changes

#### (i) Changes in intra-ocular pressure

While animals were under general anaesthetic for ERG measurements, the intra-ocular pressure was measured in the left eye with a hand-held Tono-Pen (Applanation Tonometer, Reichert Technologies, Seefeld, Germany) on seven animals of winter and eight of summer. Five consecutive measurements were made in each eye and averaged. At this stage, measurements were made of pupil diameter in both the fully dilated left eye and the fellow eye under direct illumination. This was undertaken in four animals to provide a measure of the full dynamic range of the pupil. Pupil diameter can be related to intra-ocular pressure [5].

#### (ii) *In vitro* changes in pressure on tapetum lucidum

TL was dissected from animals killed in summer, as above, and the preparations containing the TL and underlying choroid were wet mounted on slides. These were all photographed directly, and then had a pressure applied similar to the intra-ocular pressure found in winter (approx. 22 mmHg). Changes in reflection were recorded by simple observation and raw photography. This was undertaken repeatedly in two separate specimens of central TL.

## 3. Results

### (a) Seasonal changes tapetum lucidum reflectance

**Figure 1.**
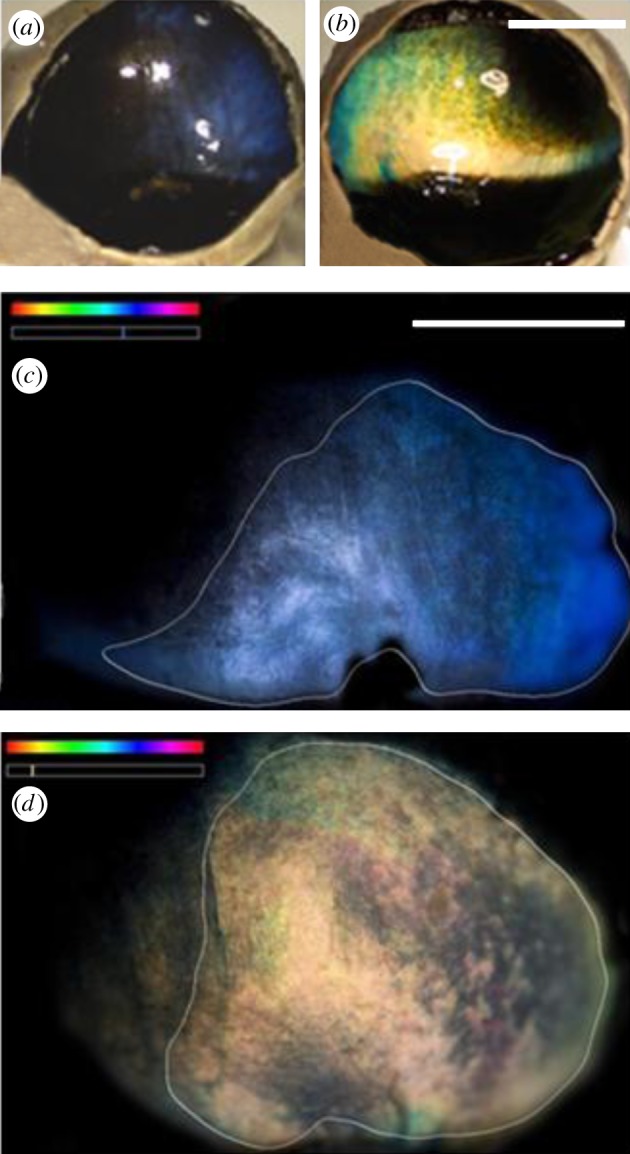
TL in (*a*) winter and (*b*) summer eyes following removal of the cornea, lens and vitreous. The winter eye was deep blue and the summer eye was golden with elements of turquoise around the edges. (*c*,*d*) Example of preparations where the eye cups have been flattened to reveal the TL in whole mount. These were used for the analysis for hue and saturation (figure 2). Mean wavelength of the reflection for (*c*) and (*d*) is given against the visible spectrum in the upper left. Scale bars, 1 cm.

**Figure 2.**
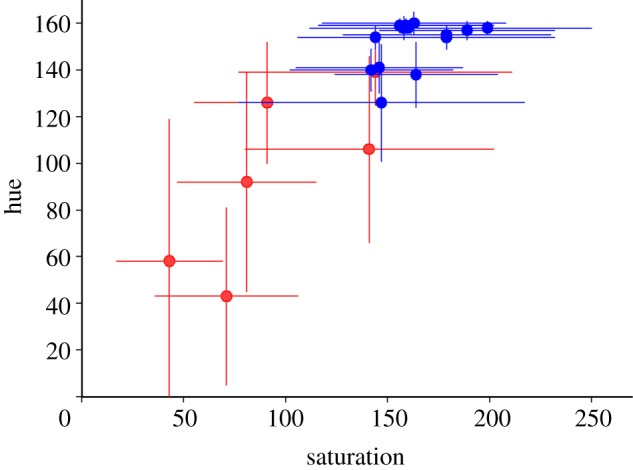
Measurements of hue against saturation for each of six summer and 14 winter eyes. The two groups were significantly different (*p* < 0.05), with summer eyes (red) in the lower left and winter eyes (blue) in the upper right. The bidirectional error bars are standard deviations (s.d.). The winter eyes are tightly grouped reflecting their more consistent blue pattern of reflection, whereas the summer eyes were more variable because they contained turquoise elements.

There were clear differences in the colour of the TL between animals killed in summer and winter. In summer, the TL was similar to that reported for other ungulates, being predominately golden [3,4]. However, this was markedly different in winter, when it was deep blue (figure 1). Colour differences were consistent between animals of the two seasons and were quantified showing clear separation between summer and winter groups both in terms of hue and saturation of the reflection (*p* < 0.01, Mann–Whitney *U*-test; figure 2). The distributions shown in figure 2 are wider for summer animals as the TL during this season contains elements of both golden and a slight turquoise colour, while the winter reflection is a more consistent deep blue. The winter blue was also apparent in eye cups examined directly after death, but prior to fixation (data not shown).

**Figure 3.**
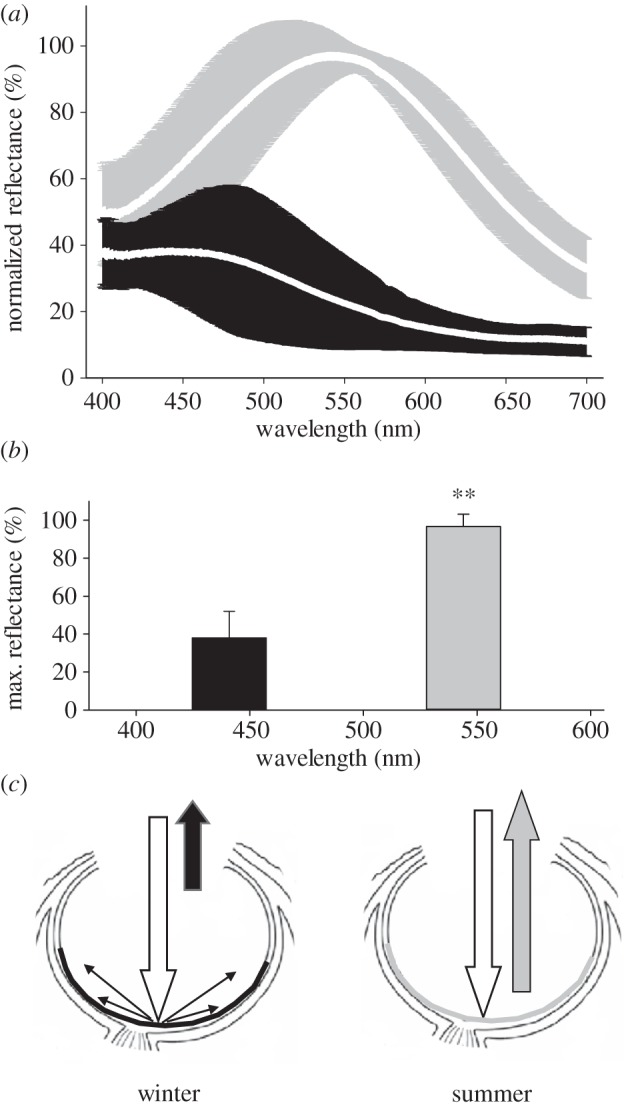
(*a*) Reflectance spectra from 400 to 700 nm from five summer (grey) and five winter (black) eyes, presented as percentage of the total reflectance against a white standard. The means are the white lines within the grey and black representation of the s.d. (*b*) The maximum reflectance for summer and winter eyes in terms of the mean peak values and s.d. shown in (*a*). Total reflectance at these wavelengths (444 nm in winter and 541 nm in summer) was significantly different (*p* < 0.001). (*c*) Panels (*a*) and (*b*) reveal that less light is reflected back from the winter eyes. Hence, this schematic figure represents how the reflectances differ between seasons. In summer, the light is reflected back spectrally from the surface of the TL, whereas in winter less light returns, possibly because it is scattered through the outer retina.

In addition to the change in colour of reflected light, the amount of light reflected from the TL was reduced in the winter blue eyes compared with the summer eyes by approximately 60% when measured at a distance of approximately 1 cm (*p* < 0.01, Mann–Whitney *U*-test; figure 3). The measurement of reflected light is made relative to a flat white standard. Consequently, it is possible for values to exceed 100%. Hence, more than 95% of the light was reflected directly back in summer eyes, compared with only 40% in winter eyes. The difference in reflectance wavelengths (figure 3*b*) is taken from the mean peaks of the curves shown in figure 3*a*, which are at 444 and 541 nm in winter and summer, respectively. Figure 3*c* shows a schematic of the potential difference in reflective properties between the eyes in the two seasons. In summer, light reflects spectrally, whereas in winter reflected light is reduced in this experiment probably owing to internal scatter associated with shorter wavelengths. To estimate the degree of internal scatter from the TL, we placed the coaxial optic fibre closer less than 1 mm to the TL surface in summer and winter eyes and stimulated with dim 510 nm light which is close to the peak absorption of rhodopsin. Increased scatter should result in more light being detected in the sensing element of the probe. Within this experimental framework, the fraction of applied light detected was approximately 0.35 in summer and 0.9 in winter in multiple measurements. This is consistent with the notion of higher local scatter in winter.

### (b) Electroretinogram recordings of retinal function

There were marked ERG differences between summer and winter. The ERG is made up of two components, the a- and the b-wave. The a-wave is negative and photoreceptor generated and the b-wave is positive and postreceptorial. However, because of the amplification of the signal in the postreceptoral cells, the b-wave appears first and is a visual threshold measure. Further, there are two ranges for the ERG, the scotopic which is low luminance and rod mediated and photopic, which is cone mediated and high luminance. Because the focus of interest is on threshold responses, data presented focus on the scotopic range.

**Figure 4.**
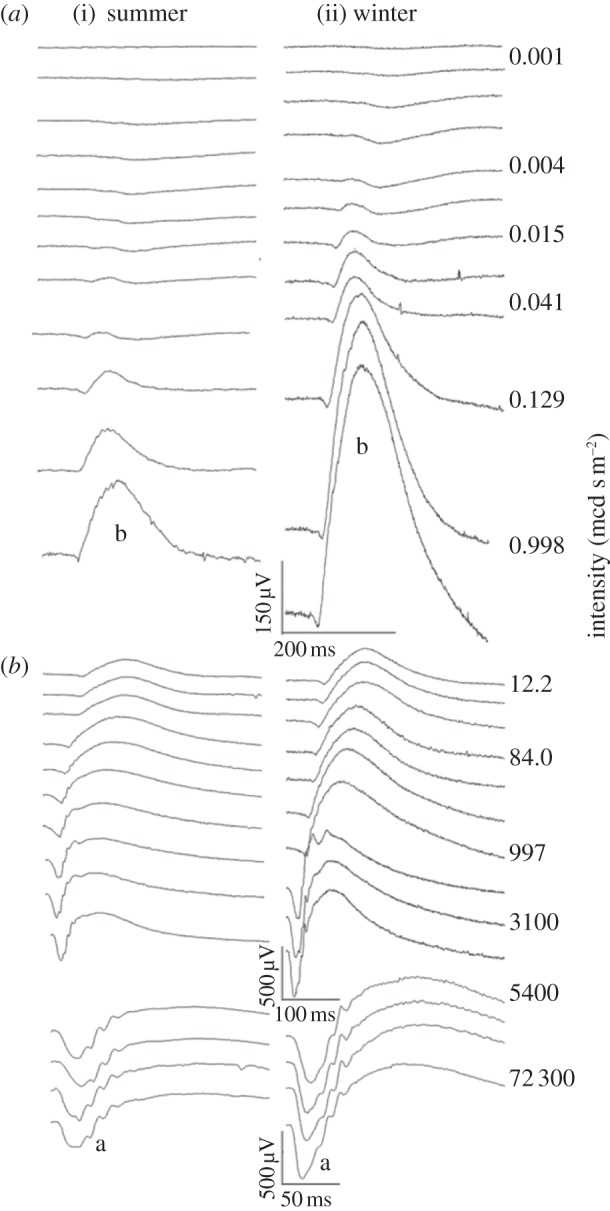
Examples of the ERG recordings from (i) summer and (ii) winter eyes for scotopic conditions. The ERG is composed of two waves, a positive b-wave (b in upper panel) and a negative a-wave (a in lower panel). As stimulus intensity increases (from top to bottom), the b-wave develops first. This wave is more than four times larger in winter eyes as apparent from the b shown in the right wave compared with the b in the left. Similarly the a-wave in the winter is more than twice the size found in summer. These differences show that the winter eye is markedly more sensitive to light over the scotopic range than the summer eye.

**Figure 5.**
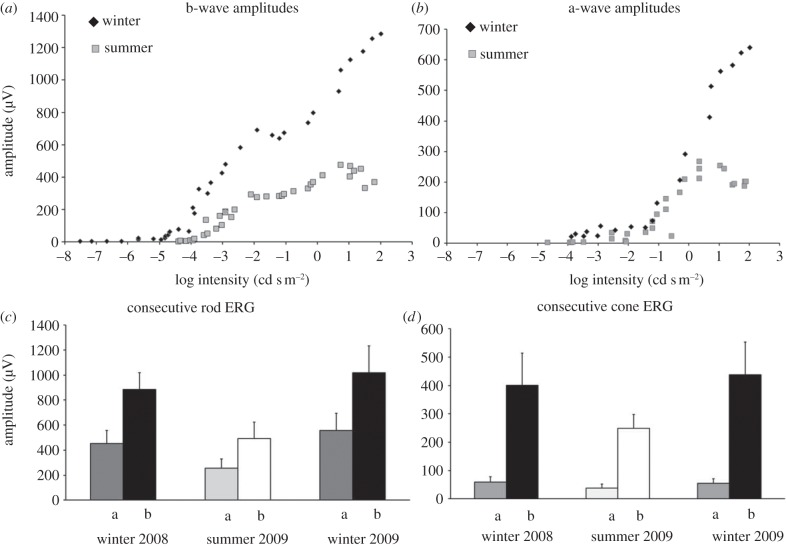
Amplitude of (*a*) scotopic b- and (*b*) a-waves over progressive stimulus intensities. (*a*) The b-wave is present in winter eyes before it becomes established in the summer animals. This is apparent for intensities between −4.5 and −7.5, where clear responses were found in winter eyes. At higher intensities, the b-wave is always greater in winter than in summer (*p* < 0.001, one-way ANOVA). In summer eyes, amplitudes asymptotes within the range were examined, but the amplitude of the winter eye is still increasing steeply at the highest stimulus intensity. (*b*) Similarly for the a-wave, amplitudes are greater in winter animals for all but the lowest stimulus intensity, and while they asymptote in summer eyes they continue to increase at the highest stimulus levels in winter eyes. (*c*,*d*) Five animals were measured repeatedly across two consecutive winters with an intervening summer for both (*c*) rod and (*d*) cone ERGs. In each case, the amplitude of winter ERGs (mean, s.e.m.) was greater than that in summer. a, a-wave; b, b-wave.

Animals examined in summer and winter had very different visual thresholds and very different responses to the same stimuli. Generally, winter animals with their blue TL had significantly elevated visual responses compared with summer animals and significantly reduced visual thresholds. Figure 4 shows scotopic ERG responses from one animal in summer and one animal in winter. The positive b-wave, which is the larger positive wave in the upper scotopic trace (*b*) is much greater in winter than summer eyes. The same is true for the negative a-wave (*a*). Figure 5*a* shows the amplitudes of the b-wave at progressive intensities, which are significantly different between seasons (*p* < 0.001, one-way ANOVA). Two key differences are apparent. First, responses obtained from winter eyes were within a range of 3 log units below threshold in summer eyes. Hence, the visual range of the animal is extended downwards in winter. Second, at every stimulus intensity examined, the amplitude of the ERG for winter eyes is greater than that for summer eyes. Hence, retina function is enhanced across the full-scotopic range. Figure 5*b* shows similar data for the a-wave. Here again, the two seasonal responses are significantly different (*p* < 0.001, one-way ANOVA**),** with those in winter eyes being greater at almost every stimulus intensity compared with summer eyes.

Responses in summer animals reach asymptote well within the stimulus range (figure 5*a*,*b*), revealing that the retinal response had saturated. Responses from winter eyes are still climbing steeply at the end of the stimulus range and the magnitudes of the responses in these eyes are likely to be greater had the stimulus intensity been increased further.

The data generated above were from single animals in single seasons. However, the change in TL reflectance shifts seasonally and a separate group of five animals were therefore tested repeatedly across two consecutive winters and an intervening summer. The results are shown for rod (figure 5*c*) and cone ERGs (figure 5*d*). Here, a-waves and b-waves are consistently greater in winter months than in the intervening summer revealing seasonal changes in the magnitude of the ERG within individual animals. The results also show that these are not confined to the rod scotopic responses, but also occur in the cone-mediated photopic responses.

### (c) Collagen spacing

**Figure 6.**
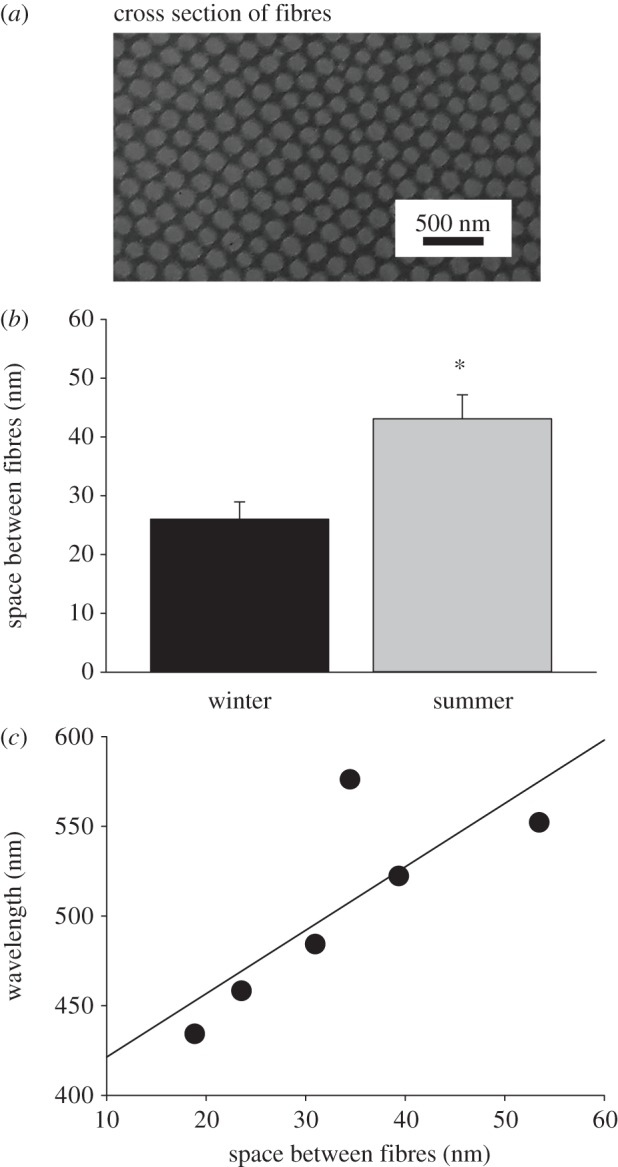
(*a*) Photomicrograph from an EM image of the TL where the collagen fibres have been sectioned across their orthogonal axis. The image reveals regularly spaced fibres. (*b*) The spacing between collagen fibres measured at the centre of the TL in eyes from four winter and four summer animals (mean, s.e.m.). Distances in winter animals are significantly smaller (26 nm) than that in summer (43.1 nm; *p < 0.05). (*c*) Six of the animals used for collagen spacing (three in summer and three in winter) were also used for reflectance measurements (figure 3). Hence, for these animals the reflected wavelengths are plotted against collagen spacing, revealing a complete separation between summer eyes (upper three) and winter eyes (lower three). The greater variability in the summer eyes is reflected in the scatter of these three points, which results in a linear regression measure falling just short of significance (*p* = 0.06).

Bragg's Law has been used in the analysis of spectral reflections from the TL [6,7]. This law relates the wavelength of reflected light to the regular spacing of elements in the reflecting surface and predicts that the closer the collagen spacing, the shorter the reflected wavelength. Figure 6*a* shows the regular spacing of collagen fibres cut orthogonally at the central TL. A clear regular matrix is apparent. Figure 6*b* shows that the spacing between collagen fibres taken from the same location in the eye in winter and summer eyes is significantly different, with that in winter being 26 nm and in summer 43.1 nm (*p* < 0.05, Mann–Whitney *U*-test). In three eyes from summer and three from winter, both reflectance and collagen distances were measured. Figure 6*c* shows wavelength reflectance against collagen spacing and reveals that there is a clear trend in these data. However, because of the low numbers and the outlier this was not significant (*r*^2^ = 0.62, *p* = 0.063). The outlier in the summer group is consistent with the greater variability found in reflectance from summer eyes (figure 2).

### (d) Pressure changes

**Figure 7.**
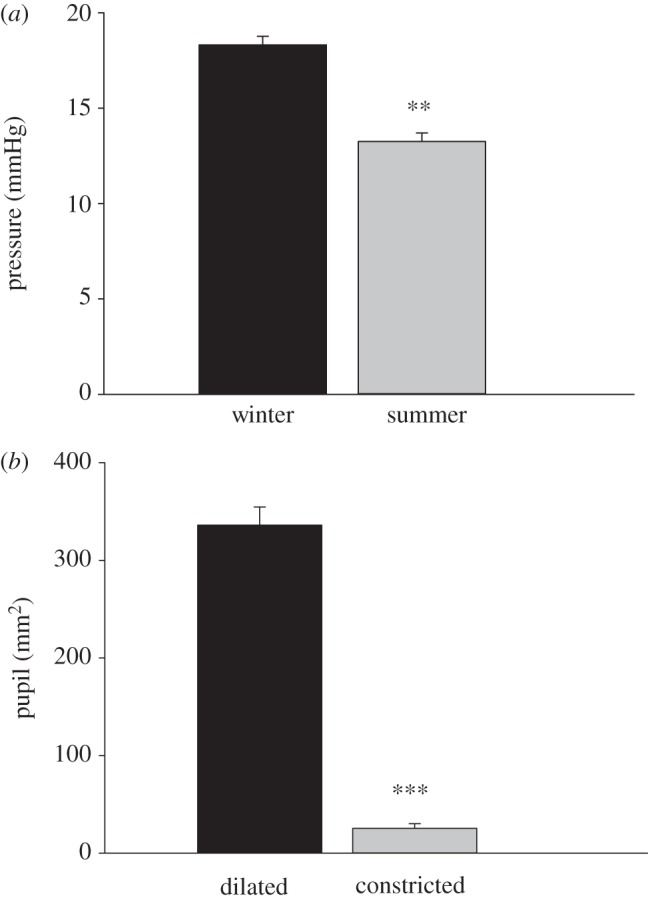
(*a*) Intra-ocular pressure in eight summer and seven winter eyes (means, s.e.m.). Pressure in winter (18.1 mmHg) was significantly larger than that in summer (13.3 mmHg; **p < 0.01). (*b*) Pupil size in four dilated eyes compared with the fellow eye under bright light. Constricted pupils were 25.3 mm^2^ and elongated horizontally. When dilated, they were 336 mm^2^ and round (***p < 0.001).

To determine whether intra-ocular pressures varied between the seasons and could be related to changes in collagen spacing, pressure measurements were taken at the end of ERG recordings. Figure 7*a* shows the intra-ocular pressures of eight summer and seven winter animals. There is a significant increase (*p* < 0.01, Mann–Whitney *U*-test) in intra-ocular pressure in winter (18.1 mmHg) compared with summer (13.3 mmHg).

Intra-ocular pressures can be related to pupil diameter because dilation results in significant elevation in pressures [8]. Constricted pupils were horizontally aligned being approximately 3 mm vertically and approximately 10 mm horizontal, having an area of about 30 mm^2^. When dilated, they were round with an area of approximately 350 mm^2^ (figure 7*b*). This difference was significant (*p* < 0.001, Mann–Whitney *U*-test)

**Figure 8.**
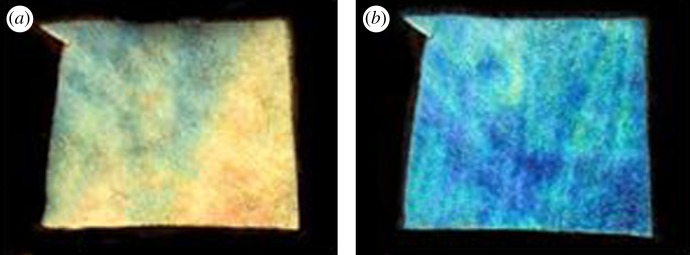
(*a*) Central TL regions from summer animals dissected free and photographed wet, revealing a golden appearance as in figure 1. (*b*) The same specimen after coverslipping with an 8 g weight applied adjacent to the specimen. This resulted in specimens turning blue, similar to the winter eyes. The area of tissue is approximately 1.7 cm^2^. Hence, increased pressure induced changes in the summer TL reflection similar to that of winter eyes.

To confirm that there is a relationship between pressure and TL reflectance, *in vitro* preparations of the summer TL were examined where pressure could be applied and reflectance viewed. Without pressure or a coverslip the TL has the normal summer appearance (figure 8). When the TL was coverslipped and an 8 g weight was applied, the reflection changed to blue immediately. Hence, the wavelength of the reflectance is related to pressure.

## 4. Discussion

This study reveals a unique mechanism of visual adaptation in a large mammal whose visual environment is challenging. At the latitude where this study was undertaken, there are two months in winter when the sun remains below the horizon and two months in summer when it remains above it. In summer, the reindeer TL appears similar to that in other ungulates, being predominantly golden [2,3]; but in winter with its extended darkness, the TL reflection shifts to deep blue with 50% less light being reflected directly back but with more light scattered through the outer retina. The shift in wavelength is associated with a reduction in the spacing of the collagen fibres that constitute the TL. It is further associated with an increase in intra-ocular pressure in winter when the pupil remains fully dilated. Linked with these winter changes is a marked increase in retinal sensitivity.

This is, to our knowledge, the first description of a seasonal change in the TL in a mammal. Also, it is highly unusual in describing a deep blue TL. Johnson [3,4] in his extensive documentation of the TL in 50 different mammals comments that the absence of blue is remarkable and that in no case did he see a uniform blue fundus. Only one mammal, the mongoose (*Herpestes griseus*), had significant blue elements. However, Ollivier *et al*. [9] noted that blue elements were present in the bovine TL, but this was not dark or uniform as in the reindeer winter eye.

Intuitively, the reduced reflection from the dark blue winter TL may be thought of arising from its surface absorbing light. But the increased retinal sensitivity revealed by the ERG argues against this and there appears to be increased scatter particularly at the wavelengths to which rhodopsin is sensitive. Hence, an obvious explanation is that blue shift increases scatter of reflected light such that the rays pass tangentially through photoreceptor outer segments rather than returning spectrally. This increases retinal sensitivity but at the cost of acuity, which may be an advantageous winter trade-off.

From an ecological perspective, the animal's key concern is to forage effectively without being predated. Because of an animal's need to use central vision for tasks such as navigation and foraging, the avoidance of predation necessarily relies on the peripheral visual field. Whereas visual camouflage can greatly reduce the visibility of a static predator, it is much more difficult for an animal to conceal its presence when moving. Various species including primates [10], fishes [11] and even invertebrates [12] are adept at detecting the spatio-temporal correlations in both luminance (first order motion) and in higher order statistics, for example local contrast (second order motion) that accompany object movement, no matter how well matched to its background at rest. In mammals, there are thought to be two fundamental visual pathways into the brain [13]. Motion detection is thought to be driven by the magnocelluar pathway sensitive to low spatial frequencies but high temporal frequencies. This is in contrast to the parvocellular pathway, which is relatively sensitive to higher spatial frequencies making it more useful for form perception. Magnocellular cells in the primate geniculate receive a disproportionately large projection from the peripheral retina, which is thought to confer high motion sensitivity in peripheral visual fields [13]. While elevated ERGs are consistent with increased photon capture and higher sensitivity it is at the cost of acuity if it is the result of scatter. But the functional separation of motion and form processing means that acuity loss will not be greatly detrimental to motion perception. Consequently, this will not carry a selective disadvantage in respect to predation.

The wavelength reflected from a fibrous surface partly depends on the distance between its ordered elements. This is known as Bragg's Law. It explains why the more tightly spaced fibres in winter reflected light at a shorter wavelength than the more widely spaced fibres in summer. Hence, changes in collagen spacing underpin the reflected wavelength from the TL. The mechanism behind this is likely to be linked to the raised intra-ocular pressure found in winter which may reduce the matrix between fibres bringing them closer together. Evidence in favour of this comes from the *in vitro* experiments where externally applied pressure was used on isolated TL segments to turn them from summer golden into winter blue. However, it also comes from Roberts *et al*. [14] who demonstrated that raised intra-ocular pressure clearly results in a reduction in choroidal thickness. Consequently, it is likely that raised pressure will act in a similar way on the TL, which is between the choroid and the site of the raised pressure.

The key source of increased intra-ocular pressure is most likely to be a blockage of aqueous flow from the anterior chamber at the trabecular meshwork. The dynamics of this in deer are similar to man [15]. It is known that dilation of the pupils can result in raised intra-ocular pressure in normal subjects and those with glaucoma [8]. Here, the impact of pupil dilation depends on the geometry of the iris and cornea. If the angle between these is relatively closed, pupil dilation and the folding back of the iris will cover the trabeclular meshwork. The anatomy of this region in the reindeer has yet to be investigated between seasons, but during extended dark winter months the reindeer pupil will be fully dilated. Our data show that the pupil dilates from approximately 25 mm^2^ to approximately 336 mm^2^, taking it from a horizontally elongated opening to round. Hence, it is likely that the mechanism driving increased pressure in winter is partial or complete blockage of the trabecular meshwork by the iris folding over it. The pressures obtained here for summer animals of approximately 13 mmHg are similar to those found in other members of the cervidae family [16]. Hence, raised winter pressure of about 18 mmHg is a seasonal deviation from the normal pattern.

Evidence in favour of a mechanism of TL reflectance changes depending on pupil dilation comes from a separate group of reindeer not included here. The present winter animals came from a remote island 80 km from Tromsø and were brought directly into the experimental environment. A separate group of animals were maintained throughout the winter at the University of Tromsø, which overlooks the town and is exposed to permanent distant urban lighting and its reflection from clouds. Their TL did not change to a deep blue in winter, but acquired a green appearance, which is a transition colour between the summer golden and the winter blue. Moreover, ERG measurements in these animals resulted in b-wave amplitudes around 800 μV at the highest stimulus intensities, which is also intermediate between summer and winter animals reported here (figure 5*a*). One possible interpretation is that exposure to distant light resulted in incomplete pupil dilation, and consequently the intra-ocular pressure did not increase to the same extent as found in the wild, but sufficient enough to result in a shift from gold to green and a partial increase in retinal sensitivity. Unfortunately, intra-ocular pressure measurements were not made in these animals.

One potentially perplexing finding is that fresh winter blue eyes did not turn gold when opened and intra-ocular pressure dropped. While we confirmed that the blue reflection was not the result of fixation, we did not examine unfixed winter preparations over extended periods to see whether there were changes over time. It is possible that had the tissue been maintained fresh, the reflection would have changed. However, immediate changes were found in the summer *in vitro* fixed preparations with applied pressure resulting in changes to blue. These only changed back slowly towards a golden colour when the weight was removed. It is probable that pressure removed fluid between fibres rapidly but that it took longer for it to diffuse back into the tissue.

One alternative explanation for improved retinal sensitivity in winter is photostasis. Here, it is argued that rhodopsin levels and outer segment lengths are adjusted by ambient lighting, with prolonged darkness producing longer segments and increases rhodopsin levels [17–19]. However, these studies were undertaken on albinos, which are ocular mutants with diverse deficits including those of outer segments [20, 21]. Further, Cunea *et al*. [22] recently questioned the notion of photostasis measuring large numbers of outer segments in different light environments and found no significant evidence for it. Critically, we also show changes in the magnitude of cone responses between seasons, but there has been no suggestion of photostasis in cone photoreceptors.

While this study has revealed a series of changes in the winter reindeer eye that are highly adaptive, it is important to stress that we have not proved functional relationships. It is likely that pupil dilation raises intra-ocular pressure and that this changes the reflectance of the TL resulting in increased retinal sensitivity. But we have not performed the conclusive experiments proving this. In part this is owing to problems of dealing with very large mammals. A key demonstration would be to dilate the summer reindeer eye for long periods and repeat some of our observations. But daily capture and restraint over months is not feasible and would be highly stressful for animals and experimenters alike.

In spite of this, the most parsimonious explanation is that these factors are functionally linked to provide an adaptive mechanism to a challenging environmental light that is completely novel among mammals. It remains to be seen whether other Arctic mammals have adopted similar strategies.

## Notes

Ethical approval for all *in vivo* experiments was obtained from The Norwegian Animal Research Authority.
